# The influence of relationships on personhood in dementia care: a qualitative, hermeneutic study

**DOI:** 10.1186/1472-6955-12-29

**Published:** 2013-12-20

**Authors:** Kari Lislerud Smebye, Marit Kirkevold

**Affiliations:** 1 Faculty of Health and Social Work Studies, Ostfold University College, 1757 Halden, Norway; 2 Institute for Health and Society, Faculty of Medicine, University of Oslo, Blindern, P.B. 1103, 0318 Oslo, Norway; 3 Institute of Public Health, Aarhus University, Aarhus, Denmark

**Keywords:** Dementia, Relationships, Personhood, Person-centred care

## Abstract

**Background:**

In dementia personhood can be understood as increasingly concealed rather than lost. The sense of being a person evolves in relationships with others. The aim of this study was to increase the understanding of the nature and quality of relationships between persons with dementia, family carers and professional caregivers and how these relationships influenced personhood in people with dementia.

**Methods:**

This Norwegian study had a qualitative hermeneutical design based on ten cases. Each case consisted of a triad: the person with dementia, the family carer and the professional caregiver. Inclusion criteria for persons with dementia were (1) 67 years or older (2) diagnosed with dementia (3) Clinical Dementia Rating score 2 ie. moderate dementia (4) able to communicate verbally.

A semi-structured interview guide was used in interviews with family carers and professional caregivers. Field notes were written after participant observation of interactions between persons with dementia and professional caregivers during morning care or activities at a day care centre. Data were analysed in two steps: (1) inductive analysis with an interpretive approach and (2) deductive analysis, applying a theoretical framework for person-centred care.

**Results:**

Relationships that sustained personhood were close emotional bonds between family carers and persons with dementia and professional relationships between caregivers and persons with dementia.

Relationships that diminished personhood were task-centred relationships and reluctant helping relationships between family carers and persons with dementia and unprofessional relationships between caregivers and persons with dementia.

**Conclusions:**

A broad range of relationships was identified. Understanding the complex nature and quality of these relationships added insight as to how they influenced the provision of care and the personhood of persons with dementia. Personhood was not only bestowed upon them by family carers and professional caregivers; they themselves were active agents who gained a sense of self by what they said and did.

## Background

The concept of personhood, understood as the quality or condition of being a person, has generated considerable debate. Western philosophers like Descartes and Locke defined a living creature as a person by their capacity for rational thinking and having memory. Within a hierarchy of attributes, these cognitive attributes were valued most [1,2]. Having continuity of memory has been associated with identity [3]. With the progressive decline in rational thinking as a result of dementia, consciousness of thinking becomes less evident and memory is affected. As dementia destroys the brain, it also destroys the person. Persons are stripped of their personhood, leading to a “loss of self” [4].

This does not correspond with studies that have documented that there is evidence for persistence of self in mild, moderate to severe stages of the illness although many studies recorded some degree of deterioration in aspects of self and identity [5-7]. Even in persons with severe dementia, episodes of lucidity revealing selfhood have been reported [8].

Kitwood reconceptualised personhood by not linking it exclusively to cognitive functioning but understood it as socially constructed in an interactional environment. He defined personhood as:

*“..a standing or status bestowed upon one human being by others in the context of particular social relationships and institutional arrangements. It implies recognition, respect and trust.”*[9]:7]

Kitwood challenged the prevailing reductionist bio-medical view of dementia by postulating that seeing the person, not just the disease, was important. Instead of persons being defined by their disease, he viewed them as basically persons and the disease as only one aspect of their lives. To be a person is to have a certain status; the intrinsic value of individuals as unique human beings makes them worthy of respect and dignity [10].

Personhood is the right of every human being regardless of capacity and it is through relationships with others that a full sense of being a person evolves. Buber postulated that all real living is meeting with mutual acknowledgement of the uniqueness of the other [11]. Personhood is thus a product of relationships with others and can be nurtured or diminished, depending on whether the person is being valued or depersonalised.

Kitwood theorised that some of the deterioration seen in people with dementia was caused not by the disease itself, but by how persons were treated. “Malignant social psychology” exists in relationships which devalue, dehumanize and diminish the person with dementia and for example, when the person is stigmatised, infantilised, objectified or ignored, a loss of personhood ensues.

An alternative approach is “positive person work” or person-centred care that aims at restoring and sustaining personhood. The underlying humanistic philosophy acknowledges that the individual is a person that can experience life and relationships, despite the progressive disease, focusing on strengths rather than on deficits [9,12]. There is no consensus on the definition of person-centred care and it can be understood as a value base, individualised care, a set of techniques or a phenomenological approach [12,13]. However, Edvardsson and colleagues [14]:363] have summarized person-centred care as having the following components:

• *Regard personhood in people with Alzheimer’s disease as increasingly concealed rather than lost*

• *Acknowledge the personhood of people with Alzheimer’s disease in all aspects of care*

• *Personalise care and surroundings*

• *Offer shared decision making*

• *Interpret behaviour from the person’s viewpoint*

• *Prioritise the relationship to the same extent as the care tasks*

According to Brooker [12]:16] the primary outcome of person-centred care for people with dementia is to maintain their personhood in the face of declining mental powers. Brooker builds on Kitwood’s work and emphasizes the importance of a caring culture that maintains personhood. A culture of care contains four major elements and can be expressed in the following equation [Brooker [12]:13]:

$$\mathrm{PCC}\;\left(\mathrm{person}-\mathrm{centred}\;\mathrm{care}\right)=V+I+P+S$$

*V - A* ***V*** *alue base that asserts the absolute value of all human lives regardless of age or cognitive ability*

*I - An* ***I*** *ndividualised approach, recognizing uniqueness*

*P - Understanding the world from the* ***P*** *erspective of the service user*

*S - Providing a* ***S*** *ocial environment that supports psychological needs*

Personhood as a concept has brought the person with dementia to the foreground but has not promoted the vision of someone with agency capable of exerting power and influencing their life situation [15]. Archer [16] underlined the “primary of practice” meaning that persons are proactive and gaining a sense of self by what they say and do. They initiate interaction and are not only influenced by how other people behave towards them. An example of how persons with dementia act as agents has been documented in a study by Smebye et al. that explored how they participated in decision making in daily care and health matters [17].

According to Kitwood [9], personhood is conferred upon a person, conveying a unidirectional or one-way understanding which continues to position a person with dementia as passively dependent on others for confirmation. Consequently, family carers and professional caregivers are responsible for sustaining the personhood of people with dementia but they can also be blamed for their mental decline [18]. When Kitwood described how persons with dementia are exposed to “malignant social psychology”, there was no reference to the agency of people with dementia. On the contrary, they were depicted as passive recipients of external forces mainly within an institutional setting as family carers were not a primary focus in Kitwood’s work [19].

Nolan et al. [20]:203] argued that person-centred care fails to *“…capture the interdependencies and reciprocities that underpin caring relationships”* and it does not elicit *“…mutual appreciation of each other’s knowledge, recognition of its equal worth, and its sharing in a symbolic way to enhance and facilitate joint understanding”.* Therefore, person-centred care needs to be expanded to “relationship-centred care” [21]. Brooker [12] claims that person-centred care takes place within the context of relationships, although it is not clear how the VIPS model takes mutuality and reciprocity in interactions into account.

According to Snyder [22] and Lawrence [23], relationships in dementia care remain the overlooked variable in many studies, with very few having explored the dynamics between the parties involved. Fortinsky [24] recommends furthering the development of health care triads in dementia care and exploring the perspectives of all participants simultaneously.

In general the literature underlines the importance of relationships but there is a paucity of theoretically and empirically rigorous studies that have made relationships the main focus of enquiry [25-27] and what they mean for the personhood of people with dementia. Bowers studied how nursing home residents defined quality of care and found that they emphasized care-as-relating with affective aspects of care as central to good care [28].

However, in a rare study Wilson et al. [25] explored the nature of relationships between residents, staff and family members in nursing homes. Three types of positive relationships were identified: pragmatic relationships; personal and responsive relationships; reciprocal relationships. Care routines were often the starting point for the development of relationships as this was a legitimate focus for interaction.

Research exploring how relationships are defined and measured is only in an early stage of development [29]. Studies that evaluate person-centred care are often small scale, in an institutional setting and include interventions with many components [30-32], making it difficult to draw solid and trustworthy conclusions [14,26,33].

The aim of this study was to increase the understanding of the nature and quality of relationships between persons with dementia, family carers and professional caregivers and how these relationships influenced personhood in people with dementia.

## Methods

The study had a qualitative, hermeneutic design [34-36] and was based on ten cases. Each case consisted of a triad: the person with dementia, the family carer and the professional caregiver, altogether thirty participants.

### Recruitment

This Norwegian study was conducted in the Eastern part of the country; in one rural and two urban municipalities. Inclusion criteria for people with dementia were: (1) 67 years or older (2) diagnosed with dementia (3) Clinical Dementia Rating [37] score 2 i.e. moderate dementia; (4) able to communicate verbally. Age 67 was chosen because this is the common retirement age in Norway.

Staff in the three municipalities were informed about the study and asked to identify persons meeting inclusion criteria. These persons were given written information by the staff letting them know that participation was voluntary, that they could withdraw at any time and their anonymity was assured. The professional caregiver who cared for them on a regular basis then asked if they were willing to participate in the study and written consent was obtained. Even though they might have felt obliged to consent when asked by a caregiver on whom they were dependent, we judged that it was better that they were asked by a known and trusted person as this is known to reduce anxiety [38,39]. Family carers were similarly given written information and asked to consent to their own participation as well as to the participation of the person with dementia. Next, informed consent was obtained from the professional caregiver of each person with dementia. On the day persons with dementia were observed, they were asked again if they still consented to participate. It was agreed that if verbal or non-verbal expressions of discomfort were registered, observations would be stopped. None of the persons with dementia showed any signs of discomfort.

The research project was approved by the Regional Committee for Medical Research Ethics in Norway – SE (reference number S-07181a) and the Norwegian Social Science Data Services (project number 17352).

### Sample

Diversity was promoted through purposive sampling. Three persons lived independently, three persons lived with close relatives and four persons had moved to sheltered housing or to a nursing home. The mean age was 83 years and two were men. The group of family carers consisted of three spouses, two siblings, three adult children, a daughter-in-law and a niece. Four family caregivers were men. The professional caregivers consisted of two registered nurses, six enrolled nurses and two nurse assistants, all women.

Twenty-three older people were asked to participate in the study of which 10 consented. The main reasons for not being included were: no diagnosis (2), did not wish to participate (8) or their family thought it would be too stressful for them (3).

### Data collection

A semi-structured interview guide with open-ended questions was used in interviews with family carers and professional caregivers. They were asked to express how they felt about their relationship with the person with dementia, how they influenced decisions about health care and their experiences of collaboration and coordination of services. The interviews lasted approximately one hour and were audio-recorded and transcribed verbatim. Field notes were written after participant observation of interactions between persons with dementia and professional caregivers during morning care or activities at a day centre. How the professional caregivers related to the person with dementia and facilitated participation in activities was the focus of the observations that lasted approximately one to two hours. Field notes were usually written immediately after the observations but also during observations when dialogues were of special interest. Sometimes the observations lasted longer when the researcher (KLS) talked to the persons with dementia, shared a meal or took part in activities (up to 4 hours extra). General impressions and reflections were recorded afterwards as recommended by Fangen [40]. Interactions between the persons with dementia and their family carers were not observed during morning care as this would have been an unacceptable invasion of their privacy. Because of the dementia trajectory, all data in each case were collected in the course of one to two days. Data were collected from October 2007 to January 2009. All interviews and observations were undertaken by one researcher (KLS) for consistency.

### Data analysis

The hermeneutic analysis [34,35] was undertaken in two main steps. The first inductive analysis allowed for different types of relationships to emerge from the data. The text from the interviews and field notes was read thoroughly to identify preliminary themes referring to types of relationships. Several in-depth readings gave a sense of the whole. A summary of each case was written.

Then text units were identified by colour coding and condensed so that patterns and themes emerged [41-43]. Each case was analysed before comparing and contrasting different types of relationships in a cross-case analysis [44] In this way the text was freed from the original context, making it easier to explore manifest and latent meanings. The analysis continued by reviewing the text from parts to the whole and back again until themes were clarified [45-47]. The first analysis generated three types of relationships between family carers and persons with dementia (close emotional bonds, task-centred relationships, reluctant helping relationships) and two types of relationships between professional caregivers and persons with dementia (professional and unprofessional relationships).

In the first analysis it was difficult to discern how the different relationships were related to personhood among the persons with dementia. In the second step the data were therefore analysed deductively, applying the theoretical framework of person-centred care, using the VIPS-criteria as described by Brooker [12]. This allowed for a more systematic analysis in order to gain a deeper understanding of the complex nature of caring relationships and how they influenced personhood. The interactions and care given by family carers and professional caregivers were analysed in this manner. For examples see Table 1 and Table 2.

**Table 1:** Analysis of case Mrs I: Relationship between family carer (FC) and person with dementia (PWD)

**VIPS - criteria**	**Data**
**V**aluing the person	Expressed appreciation of what the PWD had meant to her and done for her through the years
	Spoke to the PWD in a respectful manner
**I**ndividualised care	Gave individualised care and anticipated needs
	Paid attention to details that were important to the PWD
**P**erspective of PWD	Tried to understand feelings and reactions
	Consulted her before making decisions and respected her values and preferences
**S**ocial environment	Helped PWD to relate to significant others
	Arranged social gatherings and went on holidays together

Conclusion: Close family bonds and person-centred care enhanced the personhood of the person with dementia.

**Table 2:** Analysis of case Mrs I: Relationship between professional caregiver (PC) and person with dementia (PWD)

**VIPS - criteria**	**Data**
**V**aluing the person	Had no biographical knowledge of the PWD
Treated the PWD with respect but the PC said that this was difficult at times. When she came to help the PWD in the mornings and found her lying fully dressed on her bed, she did not offer to help her shower. In her opinion this would have been disrespectful because it would have reflected that she did not believe the PWD when she said she was done showering.	
**I**ndividualised approach	Focused on physical needs, gave individualised care
**P**erspective of the PWD	PC had no knowledge of dementia and did not attempt to understand the subjective experience of the PWD
**S**ocial environment	No time to consider psycho-social needs because of a heavy workload
Had only met FC twice in two years	
	No contact between PC in the home nursing services and staff at the day centre

Conclusion: Unprofessional relationship. Although the PC individualised the physical care that was given, other elements (V, P and S) in person-centred care were overlooked and thus personhood was diminished.

### Trustworthiness

The concepts of credibility, dependability and transferability describe the various aspects of trustworthiness addressed in this study [48]. Credibility was strengthened by continually focusing on the research question, especially during collection and analysis of data [49]. Purposeful sampling ensured that persons with dementia in various care settings were included in the study, contributing to a greater variation of caring relationships being studied. Qualitative interviews were appropriate for data collection as the interviewees were able to give rich descriptions, shedding light on the research question. Through participant observation, non-verbal signs and details in the environment were registered, supplementing data from interviews. Interviews and observations were conducted by the same researcher (KLS); both researchers discussed and analysed the data.

Triangulation of data added to the rigour of the study [45,50]. Different methods and multiple data sources contributed to more comprehensive descriptions of complex relationships. Participant observation may have influenced behaviour but concealed observation was ethically not an option. In actuality, the presence of the researcher did not seem to disturb the persons with dementia, family carers or professional caregivers. Pre-understandings were scrutinized as they influence how text develops during the interview and analysis [51,52]. Throughout the writing process, both authors discussed this issue repeatedly.

The open dialogue between the researchers added to the dependability of the research findings. An attempt has been made to leave a “decision trail” so that verification strategies such as methodological coherence, transparency and an analytical stance can be identified [48,53]. This gives the reader background to assess the transferability of the findings.

## Results

The findings have been sorted into two main themes: (1) Relationships that sustained personhood and (2) Relationships that undermined personhood. Vignettes that illustrate central aspects are added to aid the understanding of connections between relationships and personhood.

### Relationships that sustained personhood

A major finding was that close emotional bonds between family carers and persons with dementia and professional relationships between caregivers and persons with dementia sustained personhood. The care provided met the criteria of person-centred care as defined by Brooker [12] in the VIPS framework.

#### Close emotional bonds

Close family bonds were characterised by mutual affection, trust and respect. Family carers were committed to help ailing relatives. This was seen as an obvious duty and not an issue that was debated within the family. Family members knew each other well and had often lived with the person with dementia for many years; their life histories were intertwined and they had many shared memories. Family represented belonging, security and well-being. Caring activities were defined as a logical extension of family relationships.

Interdependence strengthened family cohesion and the norm of reciprocity was a motivating force. Some family carers said that the person with dementia had helped them in the past and now they saw it as their duty to repay the support they once had received. Role reversal could none the less be a challenge and required adjustments in relationships.

Family carers had unique opportunities to give person-centred care as defined by Brooker [12]. Their specific knowledge of the person with dementia enabled them to value their personal characteristics and past and present achievements; to individualise care; consider their perspectives and to maintain relationships. The following case illustrates close emotional bonds and person-centred care within a family:

Some years ago Mrs I had helped her daughter K, a single parent, with her young children. Now it was the daughter’s turn to help her widowed mother and she did so willingly. Mrs I wished to remain living in the home where she had lived with her husband for over fifty years. After a respite stay at a nursing home, Mrs I was very happy to be back home and her daughter said that when she walked into her own living room “….*she became herself again*”. There and then the daughter decided that she would do her outmost to support her mother living there by giving practical help as well as meeting her psycho-social needs. She also made sure she had regular medical check-ups and negotiated with professional caregivers when this was necessary.

The daughter’s presence made Mrs. I feel secure as illustrated in the following quote:

Not long ago I fetched her because I was taking her to the doctor and she is sitting there (in the car) and she says: “K.” I answered: “Yes.” and she continued: “Is it you?” I believe she knows there is something familiar about me and she feels secure when I am around.

The daughter respected her mother’s wish of living independently and went to great lengths to help her be in familiar surroundings that reminded her of whom she was. The daughter also helped her keep in touch with significant people in her life and continue these relationships. As often as she could she brought her youngest child along to visit because Mrs I “… *adored him more than anyone on earth*”. She also arranged for Mrs I to meet her siblings and visit the church where they were members.

#### Professional relationships

In these relationships the professional caregivers treated persons with dementia with respect and their sense of agency was promoted by caregivers who had a strengths-based approach. The focus was on remaining abilities rather than on the limiting consequences of dementia. The caregivers facilitated participation in decision making and meaningful activities by taking into account the perspective of the person with dementia, identifying their needs and giving individualised care. Risks were assessed and optimal autonomy promoted. Care tasks as well as the relationships were prioritized. They also tried to help the person keep up relationships with other significant persons in their lives. Person-centred care was delivered within the context of professional relationships that sustained personhood. This approach is illustrated in the following vignette:

There was a professional caring relationship between Mrs D and her primary caregiver. They had been neighbours for many years until Mrs D had moved to sheltered housing after her husband had died. The professional caregiver was an enrolled nurse and had 15 years of experience working in the home nursing services. When asked about what she thought was important in caring for Mrs. D she replied:

When I come in the morning I greet her and I try to listen to her tone of voice – if she is happy or tired or whatever – I can hear this right away. She is usually in a good mood but sometimes if she is feeling a bit out of shape, I can hear it right away….

She can be very aggressive. She doesn’t like everybody and neither does she cooperate well with them … I think it has to do with chemistry. But when it comes to me, strangely enough, it has not been a problem, maybe because I have known her all my life. Sometimes she talks incoherently…, therefore you must know her life-history to be able to understand and be able to ask direct questions. This is a woman you both saw and heard, she always talked loudly and stated her opinion clearly – and still does….

…..and I learnt from her husband to inform her in advance. By preparing her for what was coming, she could predict what would happen next so that things didn’t happen unexpectedly. For example he could say: “Put on your nightgown because it is soon bedtime.”

I have also found that it is absolutely necessary that I decide that her personal hygiene must be carried out and that she must put on clean underwear. I feel it has to do with her dignity too; I try to preserve her self-esteem and not “wash” it away. I try to wash her in a dignified manner even though you cannot avoid her becoming aggressive at times.

Sometimes you have to understand that she is having a hard time and then I try to divert her attention. After morning care we usually sit down together and I help her to “gather loose ends” and enjoy a quiet moment. Feeling secure has a lot to do with optimal functioning. (In the field notes it is recorded that they sat on the bed whilst Mrs D chose and talked about jewellery that matched what she was wearing that day.)

Showing respect and preserving dignity were major concerns for the professional caregiver and she took this into consideration in the manner she carried out tasks. In addition knowledge of Mrs D aided communication between them. At times she had to reflect on ethical dilemmas such as patient autonomy versus paternalism. She knew Mrs D and let her knowledge of dementia guide her actions whilst continually trying to understand and take her perspective. Individualised care was important and she took care of details that Mrs D appreciated. The professional caregiver was attentive to the husband’s advice on how to treat Mrs D and acknowledged his contributions to how she could give person-centred care.

Another example of professional caregiver relationships and person-centred care that enhanced personhood was seen in the case with Mrs J. In contrast to the home situation where Mrs J withdrew from difficult situations with her husband, she enjoyed being at the day centre where she was described as a cheerful and sociable woman. Since she was relatively younger and fitter than the others, she was able to help them and their appreciation pleased her. The staff showed their respect for her by listening to her accounts of life with her husband and tried to understand her perspectives. They tailored activities according to her interests and she took pride in mastering these tasks. Neither outbursts of bad temper nor episodes of incontinence (which was a constant problem at home) occurred at the day centre. The caregivers demonstrated person-centred care building on the elements of respect, individualised care, taking the person’s perspective and providing a social environment.

### Relationships that diminished personhood

Reluctant helping relationships or task-centred relationships between the family carers and persons with dementia diminished personhood. Unprofessional relationships between caregivers and persons with dementia had the same effect. The care provided did not meet the criteria of person-centred care as defined by Brooker [12] in the VIPS framework.

#### Reluctant helping relationship

In reluctant helping relationships family carers were hesitant to take responsibility for a relative with dementia, being primarily motivated by a sense of duty and obligation rather than love and affection. Living up to cultural values and societal expectations was important to them. Another characteristic was that the caring relationship had lasted for many years, leaving family carers tired and worn-out. The carers’ own needs were neglected and future plans thwarted, leading to frustration and disappointment. The previous nature of the relationships influenced present relationships. This was especially evident in strained relationships with long-lasting conflicts, lack of reciprocity and power-struggles between spouses. This resulted in tensions, bitterness and malignant social psychology. At times family carers were embarrassed by deviant behaviour and attributed this to the person’s ill intensions rather than to the disease.

Mrs J had earlier been a devoted wife and mother and a gracious hostess. With dementia Mrs J became incontinent, her personal hygiene deteriorated, she did not dress appropriately and she forgot where she stored objects. Mr J had a reluctant helping relationship with his wife as illustrated by the following:

When she is at the day centre at least I don’t have to think about her. When we are home together, she follows me around like a dog. So, sometimes I have asked: “What do you want? What are you doing?” And she answers: “I’m only doing this or that.” or “Nothing!” And then she probably understands that I feel a little uncomfortable and then she just sits in the kitchen and sulks – and then she sulks the whole evening.

This was an example of malignant social psychology where communication broke down and personhood was undermined. Mr J wanted rational reasons for his wife’s behaviour but she was unable to give him satisfactory answers. She probably sensed that her behaviour annoyed her husband and she withdrew from the situation. The husband did not attempt to see the situation from his wife’s perspective and he was unable to meet her needs.

When she was at the day centre, the husband said he did not think of her, which could mean that it was a relief from the pressure of being responsible for her most of the time. As her husband he could have felt that he had no option but to help her “in sickness or health” with reference to their marriage vows. Comparing her to a dog that followed him around all the time indicated that this was experienced as stressful and limiting. The personhoods of both husband and wife were diminished in this situation.

Mr J had little contact with the staff at the day centre. He declined their offer of meeting with him and he failed to return the form for biographical information about his wife. This was not surprising if he imagined that it would lead to further involvement and he already was at the point of despair.

#### Task-centred relationships

In some cases the family carers gave instrumental help rather than directly expressing their emotions and affections for the persons with dementia. They gave practical help such as shopping and paying bills. A major task for many family carers had been accessing health services, negotiating with service providers and monitoring service quality. For many family carers, carrying out these tasks was a way of showing their concern for the person with dementia. However, these relationships did not appear to have been very close previously. In some cases, when the family carers had finally managed to access help, they then considered their job done, resulting in decreased contact between family members. Such cases were characterized by dwindling social networks with few people whom the persons with dementia could rely on for support. Their family carers gave them practical help but did little to sustain their personhood in other ways.

Mr F had a hip fracture and his wife, who had experience working in a hospital, saw it as her primary task to rehabilitate him so that he could regain his mobility. The couple visited Southern Europe regularly and their aim was to resume their travelling. In order to achieve this, she focused on his physical rehabilitation.

“You can put it this way – I don’t think he would be walking around now if I hadn’t been at him like I’ve been….I force him to train… I feel that this is the only thing I can do for him. …. I give him directions and he does as he is told …. And I remind him to eat and drink because he forgets. I told him that if he did not get up and walk, he would be left sitting there in a wheel chair. Those are his options!”

At the day centre the professional caregiver was critical of the way he was treated by his wife and said: *“There is no one here with a whip to threaten him to walk whatever the cost ….”*

Mrs F was perhaps not aware of how she influenced her husband’s personhood by the way she positioned him and interacted with him. She made great efforts to help him and did what she believed to be in his best interests. However, when Mr F was with his wife, he was not given the opportunity of participating in decision making or of being an agent in his own right. It seemed as if she hoped that her efforts would restore his physical functioning. This helped her block out the reality of his dementia so that they could resume their former life-style almost as before. She seemed too tired and despondent to accept her husband’s dementia and give him psycho-social support. The professional caregiver did not have much contact with Mrs F nor did she have the time to listen to her experiences of caring for her husband. Instead she was judgemental and compared the wife’s methods to using a whip – a tool used to punish or coerce. The professional caregiver had a heavy workload and no opportunity to develop a relationship with his wife. It appeared that Mrs F had no one else to confide in and might have needed and appreciated being understood and counselled by professional caregivers.

#### Unprofessional relationships

Some professional caregivers did the necessary “bed and body work” but did not invest in the relationship with the person with dementia. They were friendly and polite but saw their work as a practical job that had to be done. Ritualized and routine care was the rule rather than the exception. It did not matter who helped the person with dementia as long as the work got done. The allotted time to do the work was inadequate, resulting in the staff being stressed. This seemed to occur more frequently in institutions than in the home nursing services.

Some professional caregivers postulated that person-centred care was the ideal they wished to follow but that they found this difficult to practice. This gap between ideals and reality could partly be explained by the work-place culture based on unclear values. Leaders did not always explicitly define and discuss the underlying nursing philosophy. Other contributing factors were repeated reorganizing of services and ensuing unclear responsibilities. Part-time and unqualified staff often with mercenary rather than affective motivations, high turn-over and inadequate documentation made matters worse. When caregivers did not work well together, unrest was passed on to residents on the ward, resulting in challenging behaviour and squabbling amongst residents (field notes). Confrontations occurred when caregivers told residents in irritated voices that they were mistaken or had done something wrong. With heavy workloads and little contact with family carers, they were less likely to ask for information on preferences, beliefs and life-history.

The following two examples illustrate unprofessional care that diminished personhood in nursing homes.

Case Miss H:

The caregiver used plastic gloves whilst washing Miss H. To save time Miss H sat on the toilet (called “the throne” by the caregiver) whilst being washed. She asked her questions as she was brushing her teeth but did not give her an opportunity to answer afterwards. When she did not understand the patient’s dialect, she did not attempt to clarify what the patient was trying to say (field notes).

In this situation several small incidents could be misunderstood by the person with dementia and result in undermining personhood. For example the caregiver could have worn gloves for hygienic reasons and was using humour when she called the toilet a ”throne”, giving her the benefit of the doubt. Yet by using gloves the caregiver could have signalled that touching Miss H with bare hands was unpleasant, hardly making her feel good about her body. Miss H was not given the opportunity to communicate adequately and combining toileting with taking care of personal hygiene was most likely seen as an efficient way of getting through morning care.

Case Mrs E:

An unqualified caregiver helped Mrs E on a regular basis. She worked part-time as other jobs were hard to find. She frequently worked extra shifts when there was a shortage of staff on the ward.

The caregiver described Mrs E as a “good” patient who did as she was told and did not make extra demands on the staff. Mrs E wished her family could visit her more often and at times she was depressed and did not sleep well because she worried about family matters. The caregiver said she did not discuss these matters with the resident as her main task was to help Mrs E with activities of daily living.

During morning care Mrs. E was very dizzy but had to stand whilst being washed. This was practical and quicker for the caregiver as there were several residents needing help before breakfast. Before leaving the bathroom, Mrs E looked in the mirror and noticed that she was not wearing her dentures.

Mrs H: “I need help to put my smile back on.”

PC: “You bungled. You mislaid your dentures long ago!”

Mrs H: “My smile has left me….”

And later…

Mrs H:” I would like a cigarette. We can go for a smoke together….”

PC: “My girl, you and I do not smoke at the same place.”

The caregiver did not attempt to understand what Mrs H meant by what she said; sometimes there were double meanings that she ignored. She also belittled her in the way she addressed her. By stating that they did not smoke in the same area she also underlined the differences between “them” and “us” and did not emphasize their common worth as human beings. By limiting her understanding of care responsibilities to only encompass physical care, she could be distancing herself and thereby protecting herself from her own feelings of helplessness. Individual needs were not documented in nursing reports and the caregiver admitted that: “*It is not so easy to remember to cut off the crusts when it isn’t written down and there are many part-time workers here. She eats more if we do…”* This was important information as Mrs E had a poor appetite and only weighed 40 kg. There was seldom contact between the caregiver and Mrs E’s son. He said he did not wish to “meddle” in the affairs of the staff as this only led to “trouble”. No biographical information was registered and since the caregiver did not know Mrs E, it was difficult for her to understand Mrs E’s frame of reference.

## Discussion

The aim of this study was to increase the understanding of the nature and quality of relationships between persons with dementia and family carers and professional caregivers and how these relationships influenced personhood.

### A broad range of relationships

In our study we identified a broad range of relationships in contrast to the study by Wilson et al. [25] that focused on positive relationships. The broad range of relationships underscores the importance of understanding how relationships between involved persons contribute to a unique context for caring. Personhood can be sustained or diminished in these different contexts.

In family relationships where personhood was sustained, past history and family coherence explained why current relationships were close. Family carers wanted to contribute and collaborated with professional caregivers for the well-being of the person with dementia. Professional relationships between caregivers and persons with dementia and their families were based on the principles of person-centred care. Reciprocity and the sharing of information were important. Professional caregivers took account of opinions and concerns expressed by persons with dementia and their family carers, and acknowledged their contributions such as sharing life-stories and using this knowledge to personalise care. On the other hand, family carers were grateful for information from caregivers about the disease, how the persons with dementia thrived and how services were organised. This in turn especially helped family carers understand the pressures of the staff and their difficult working conditions. Relationships were important for staff commitment and job satisfaction [54] and preventing burn-out [55]. According to Kitwood the personhood of carers and caregivers needed to be sustained so that they could provide person-centred care. Mutual understanding developed gradually and made it easier to work together.

In this study relationships were developed through open communication and regular and continuous interactions, consistent with a study by Sandberg et al. [56]. Wilson et al. [25] found in their study that relationships had the potential to evolve from pragmatic to reciprocal relationships by taking a relationship-centred approach to care routines. By sharing information and involvement in care decisions, caregivers were able to see beyond physical needs and also include psycho-social needs. In our study, this was demonstrated in the case of Mrs D when the caregiver took time to listen to what she said about her jewellery and what the items meant to her. In the calm atmosphere, Mrs D was lucid and talkative and the caregiver learned about the persons who had given her these gifts and on what occasions. In such episodes of lucidity the persons with dementia reveal their personhood and relationships are strengthened [8]. The caregiver was able to use this knowledge to understand Mrs D’s needs of self-affirmation and to maintain identity. Relationships are not static but are continuously evolving and further research could add to our understanding of the dynamics in relationships with the passing of time and the progression of dementia.

Relationships also need to take into account “embodied selfhood”, a concept developed by Kontos [57]. The body is the carrier of personhood and as rational abilities are reduced, meaning can also be expressed through body language [58]. The manner in which care routines are carried out is important and increased attention must be paid to bodily responses.

In cases where there were poor family relationships, past history of family conflict and discord explained why current relationships were not so close. When family carers did not relate well with persons with dementia, this reduced their interest in investing in a relationship with the professional caregiver. They either withdrew or criticized professional caregivers. In unprofessional relationships between caregivers and persons with dementia, task-centred actions prevailed over person-centred care. This aligns with studies by Hallberg et al. [59], Norberg et al. [60], Skovdahl et al. [61]. Getting through a daily timetable of practical tasks with the person with dementia in the shortest possible time was emphasized. This gave little opportunity to present a multiplicity of selves and identities. “Good” patients and family carers did not make demands on the staff and thus they could work more efficiently. For the same reasons, family involvement was not always welcomed by staff and could lead to “trouble”. As in other studies, alternative explanations could be that they considered this an indication of distrust in professional carers’ abilities or that they threatened the expertise of professional carers [62,63]. Some professional caregivers seemed to keep their distance as a coping strategy so as not to be overpowered by their own feelings of helplessness [64,65].

In relationships that sustained personhood family carers and professional caregivers collaborated and reinforced each others’ efforts. However, in this study triadic relationships sometimes led to adversarial and competitive roles and collusive alliances between two parties were formed, excluding the third party, consistent with a study by Adams and Gardiner [66]. If this was the person with dementia, this constituted a grave situation because they then became isolated from relationships that sustained personhood. In some instances compensatory relationships existed. Mrs J’s relationship with her husband did not support her personhood in contrast to what she experienced at the day centre where professional caregivers gave person-centred care.

Relationships determine the difference between “person-centred care” and “individualised care”. These terms are often used interchangeably. Individualised care focuses on tailoring care to specific individual needs and not necessarily on promoting personhood in social relationships [19]. Mrs F gave individualised care in rehabilitating her husband after his operation. However, she did not treat him with respect by not involving him in decision making, ignoring his perspectives and not understanding his psycho-social needs. Including these elements in his care would have constituted person-centred care.

#### Family carers and professional caregivers contribute different types of knowledge in caring relationships

Professional caregivers had general knowledge of dementia, the impact of the disease and how to implement helpful interventions. This knowledge consisted of facts and theories on dementia but was not contextualised and was impersonal. Knowledge of the disease was important to maintain the relationship separate from the person’s qualities and symptoms [67].

Family carers had long-lasting relationships and specific and detailed knowledge of the person with dementia, obtained through a lifetime of shared experiences. This was the foundation of their caring efforts and helped them understand the person’s emotions and needs. They could not be replaced by anyone else. Family carers were seen as having expert knowledge of the person with dementia. Professional caregivers valued this and said they had much to learn from family carers.

These findings concerning different types of knowledge are supported by Liaschenko [68] and Havarth [69]. In a study by Ward-Griffin [70], family carers and professional caregivers considered professional expertise superior to the competencies of family carers. Collaboration was equated with compliance to what professional caregivers considered the best course of action - underlining that control and power is vested with those who hold the most “prized” knowledge. It is suggested that instead of claiming that one of the parties is *the* expert, a different perspective is seeing each party as *an* expert. Mutual respect for complementing competencies lays the foundation for cooperating in holding the fragmented self of the person with dementia together [71].

In this study biographical information and life-history work was not registered systematically even though professional caregivers believed that this was important. In several instances relatives were requested to fill in information forms on their own instead of being interviewed by the professional caregiver which would have been more helpful in promoting relationships. However, this deficiency seemed to be overcome by developing good relationships through consistent staff assignments and day-to-day interactions or previous knowledge of each other. This resulted in professional caregivers having contextualised and personal knowledge of persons with dementia. Including the person with dementia in interviews for recording life history was not registered in any of the cases.

#### Personhood – bestowed on persons with dementia or gained by acting as agents?

The quality of the interactions with persons of dementia in this study depended on how they were positioned, understood and subsequently treated. In relationships that sustained personhood they were positioned as agents capable of initiating interactions and responding to other people. This finding is supported by Archer [16] who maintained that persons with dementia were agents who gained a sense of self by what they said and did. This is in contrast to Kitwood [9] who postulated that personhood was “bestowed” upon the person and accepted it passively.

Even though persons with dementia actively form and assert their personhood, some are not capable of doing so and need personhood to be bestowed on them especially in the face of progressive decline in dementia and if they do not experience relationships that support personhood. They need other people to confirm their worth and recognize who they are.

An issue that can be raised is whether close emotional bonds can lead to overprotection and doing too much for the person with dementia and thus depriving them of being agents who are able to initiate actions on their own behalf. Undermining their capacity in this way could have the effect of diminishing their personhood.

#### Relationships and quality of care

The majority of professional caregivers in this study focused on giving person-centred care, which has increasingly been advocated as a means to provide good quality care. However, there is no consensus on the definition of person-centred care and translating the theoretical framework into practice is difficult [12,13,72]. Although nurses may share the ideal of person-centred care, they doubt if it can actually be achieved because of organizational constraints and lack of resources [73-75]. In Norway, reports have been published that testify to dementia care not meeting the standards of good quality care [76-78]. This is documented in spite of specific legislation guaranteeing service users dignity and quality in care.^a,b^

One way of improving the quality of dementia care is moving away from the individualist model proposed by person-centred care and towards an understanding of relationships and interconnectedness. This adds to the understanding of what it means to be involved in a care relationship and the importance of working in partnerships. Forbat [79]:234] claims that compared to family carers, dementia care nurses

“… have no prior relationship by which their current interactions can be marked against. This can explain why a relationship-centred approach has had less emphasis than the more immediately meaningful person-centred ideas. For the family carer, however, the relationship is often at the forefront of how, and indeed why, care is delivered. For family carers, then, there is a need not just to recognise that they play an integral part in providing assistance… but to place relationships centrally within this understanding.”

Kitwood’s views on person-centred care [9] need not be dismissed but rather integrated with more recent insights, allowing for a broader view of dementia care.

#### Strengths and limitations of the study

The strength of this study is that it offers rich descriptions and interpretations of relationships in dementia care that increase our understanding of how relationships between persons with dementia, family carers and professional caregivers influence social interactions and the personhood of the person with dementia. Persons with dementia were not interviewed themselves and therefore assumptions had to be made on the basis of the collected data. Without their voice there is a void in the findings. It could have been onerous for them to be interviewed. In addition the ethics committee was restrictive in approving research involving interviews of persons where there was doubt about their ability to give informed consent.

Purposive sampling promoted diversity. The residential settings varied from living independently in their own homes to being patients in special units in nursing homes, and therefore involving different types of relationships. The small sample may have limited types of relationships that emerged. The findings cannot be generalised to apply to all persons with dementia but this study has identified the complexity of these relationships. Relationships with other family members and friends were not within the scope of this study.

## Conclusions

In this study there were a variety of relationships between persons with dementia, their family carers and professional caregivers. Understanding the complex nature and quality of these relationships add insight as to how they influence the provision of care and the personhood of the person with dementia. These perspectives need to become an integral part of dementia care. The personhood of persons with dementia who participated in the study was not only bestowed upon them by family carers and professional caregivers. They themselves were active agents who gained a sense of self by what they said and did.

## Endnotes

^a^Forskrift av 2003-06-27 nr. 792 om kvalitet i pleie- og omsorgstjenestene for tjenesteyting etter lov av 19. november 1982 nr. 66 om helsetjenesten i kommunene og etter lov av 19. desember 1991 nr.81 om sosiale tjenester m.v.

^b^Forskrift av 2010-12-11 nr. 1426 om en verdig eldreomsorg etter lov av 2011-06-24 nr. 30 om kommunale helse- og omsorgstjenester med mer.

## Competing interests

The authors declare that they have no competing interests.

## Authors’ contributions

KLS had the main responsibility of for conception and design, acquisition of data, analysis of data in addition to drafting the manuscript. MK contributed to the conception and design of the study, analysis and interpretation of data and revising the article critically. Both authors read and approved the final manuscript.

## Pre-publication history

The pre-publication history for this paper can be accessed here:

http://www.biomedcentral.com/1472-6955/12/29/prepub
